# Identification of risk factors and the pattern of lower cervical lymph node metastasis in esophageal cancer: implications for radiotherapy target delineation

**DOI:** 10.18632/oncotarget.14761

**Published:** 2017-01-19

**Authors:** Yijun Luo, Xiaoli Wang, Yuhui Liu, Chengang Wang, Yong Huang, Jinming Yu, Minghuan Li

**Affiliations:** ^1^ School of Medicine and Life Sciences, University of Jinan-Shandong Academy of Medical Sciences, Jinan, Shandong Province, China; ^2^ Department of Radiation Oncology, Shandong Cancer Hospital Affiliated to Shandong University, Jinan, Shandong Province, China; ^3^ Department of Radiology, Shandong Cancer Hospital Affiliated to Shandong University, Jinan, Shandong Province, China

**Keywords:** esophageal carcinoma, radiotherapy, target volume definition, lower cervical lymph node, risk factors

## Abstract

Radiotherapy remains the important therapeutic strategy for patients with esophageal cancer (EC). At present, there is no uniform opinion or standard care on the range of radiotherapy in the treatment of EC patients. This study aimed to investigate the risk factors associated with lower cervical lymph node metastasis (LNM) and to explore the distribution pattern of lower cervical metastatic lymph nodes. It could provide useful information regarding accurate target volume delineation for EC. We identified 239 patients who initial diagnosed with esophageal squamous cell carcinoma. The clinicopathological factors related to LNM were analyzed and the locations of the lower cervical metastatic lymph nodes were transferred onto computed tomography images. The lower cervical area was further divided into four subgroups areas. The results showed that the incidence of lower cervical LNM was 37.2 % (89 of 239) and 94.4 % (84 of 89 patients) patients had subgroup II and/or subgroup III region LNM. Of those patients, 151 nodes were considered to be clinical metastatic in the lower cervical region and 96% nodes were located in group II and group III. Based on the present study, prophylactic irradiating to lower cervical areas is recommended for patients with deeper tumor invasion, the mediastinal level 1, 2, and 4 station LNM and the more number of LNM. The atlas showed that, for the lower cervical area, the subgroup II and III region should be precisely covered in the target volume and the subgroup I and IV may be spared for minimizing the toxicity.

## INTRODUCTION

Esophageal cancer (EC) continues to be one of the most common malignancies and the sixth leading cause of cancer-related mortality over the world. [1, 2] Concurrent chemoradiotherapy (CCRT) is the standard therapy for locally advanced inoperable EC patients. [3] For the lymph node (LN) clinical target volume (CTV), historically, radiotherapy portal included all paraesophageal lymph node, including cervical or celiac nodes irrespective of initial disease extent. [4, 5] Recently, involved-field radiotherapy (IFRT) is becoming increasingly prevalent among modern studies, because it resulted in decreased irradiation toxicities without sacrificing overall survival in patients with EC. [6–9] Of note, account patients with EC of lymph node metastasis frequently occurred in paraesophageal, subclinical disease in mediastinal region may therefore have received incidental radiation dose. [10] Accordingly, although clinical practice used IFRT for patients with EC, the neighboring lymphatic regions (especially mediastinal regions) also received an incidental radiation. Nevertheless, lower cervical region rarely received incidental radiotherapy. Based on our observation, there are recent reports suggesting higher than expected nodal recurrence in the lower cervical region after IFRT for locally advanced esophageal cancer. [8, 11–13] Thus, for patients with high risk of lower cervical lymph nodes failure, elective nodal irradiation of lower cervical areas may be necessary.

For successful treatment with prophylactic node irradiation, precise delineation of the lymph node region at risk is critical. Because inappropriate delivery could potentially cause loco-regional relapse or excess toxicity. At present, there is no uniform opinion or standard on the range of radiotherapy in the treatment of esophageal cancer. Traditionally, the lower cervical nodal clinical target volume are based primarily on the distribution of normal lymphatics or by vascular and bony landmarks. Data documenting the location of involved lower cervical LNs in EC are scarce. Thus, it is an urgent need to investigate detailed involvement patterns in the lower cervical region for guiding the delineation of the target area of EC.

The aim of the retrospective study was to investigate the risk factors associated with lower cervical LN metastasis and to explore the distribution pattern of lower cervical metastatic LNs. And finally we hope to provide useful information regarding accurate target volume delineation for EC.

## RESULTS

### Patient information

In the present study, 239 patients who initial diagnosed with esophageal squamous cell carcinoma (ESCC) were enrolled. All patients did not receive anti-tumor treatment. Of the 239 patients whose records were examined, 168 are men and 71 are women, with a ratio of 2.4:1. Their age ranged from 30 to 81 years, and the median age was 59 years. Primary tumors were located in the upper thoracic esophagus in 69 patients (28.9%), the middle thoracic esophagus in 70 (56.9%), and the lower thoracic esophagus in 18 (14.2%). According to the seventh AJCC staging system for EC, in the entire cohort of patients, 11.3 % for stage I tumors, 19.2% for stage II, 55.6% for stage III, and 23.7% for stage IV tumors. The general characteristics of the enrolled patients was listed in Table 1.

**Table 1:** Univariate and multivariate analyses of the clinical factors associated with the supraclavicular lymph node metastasis

Factor	n	Supraclavicular LNM		Univariate P value	multivariate *P* value
		Yes	No		
Age					
<60	138	45	93	0.084	
>=60	101	44	57		
Gender					
Male	168	57	111	0.103	
Female	71	32	39		
Tumor location					
Upper thoracic	69	33	36	0.002	0.261
Middle thoracic	136	52	84		
lower thoracic	34	4	30		
Lesion length					
<=5cm	130	46	84	0.149	
>5cm	109	43	66		
Tumor stage					
T1-2	113	21	92	<0.0001	0.000
T3-4	126	68	58		
Level 1-2 LNM					
Yes	103	71	32	<0.0001	0.000
No	136	18	118		
Level 3 LNM					
Yes	79	36	43	0.131	
No	150	53	97		
Level 4 LNM					
Yes	107	68	39	<0.0001	0.000
No	132	21	111		
Level 5 LNM					
Yes	92	26	66	0.023	0.263
No	147	63	84		
Level 6 LNM					
Yes	46	22	24	0.247	
No	193	67	126		
Level 7 LNM					
Yes	102	32	70	0.106	
No	137	57	80		
No of LNM					
0	81	0	81	<0.0001	0.000
1-2	76	36	40		
3-6	61	38	23		
≥7	21	15	6		

Abbreviation: LNM, lymph node metastasis

### Risk factors for lower cervical lymph node metastasis

We analyzed the relationship between clinical factors and lower cervical LN metastasis. Several clinical factors were observed to be associated with lower cervical lymph nodes metastasis by univariate and multivariate analyses in Table 1. The univariate analysis showed that tumor localization, tumor invasion depth, mediastinal level 1-2 LNM, mediastinal level 4 LNM, mediastinal level 5 LNM and the number of LNM were the significant risk factors for metastasis in the lower cervical area. The multivariate logistic regression analysis demonstrated that tumor invasion depth, mediastinal level 1-2 LNM, mediastinal level 4 LNM and the number of LNM were independent risk factors for lower cervical lymph nodes metastasis. Based on these results, we recommend elective irradiation to patients with at least one of these factors.

### Location of lymph node metastasis

Our study demonstrated that the rate of lower cervical LNM was 37.2 % (89 of 239). Among those patients, lower cervical subgroup III LNM were affirmed in 67 of 89 patients (75.3 %), followed by the sequence of subgroup II lymph nodes 69.7% (62 of 89 patients), subgroup I lymph nodes 4.5% (4 of 89 patients), and subgroup IV lymph nodes 1.1% (1 of 89 patients), respectively. According to our results, 94.4 % (84 of 89 patients) had subgroup II and/or subgroup III regions LNM, while only 5 of 89 patients (5.6 %) with subgroup I and subgroup IV region LNM.

In addition, we analyzed the distribution pattern of lower cervical LNM in these patients. In the entire cohort, 151 nodes were considered to be metastatic in the lower cervical region of those patients. The median number of positive nodes was 2 (ranged, 1-5). The anatomic distribution of metastatic nodes was 4 of 151 (2.6%) in group I, 68 of 151(45%) in group II, 77 of 151 nodes (51%) in group III, and 2 of 151 (1.4%) in group IV, respectively. The distribution of 151 lower cervical nodes in different subgroup regions were listed in Table 2, and axial images demonstrating the anatomic distribution of all of these lymph nodes was shown in Figure 2.

**Table 2:** Anatomic distribution of involved lymph node in different regions

subgroup	right side	left side	Total nodes (%)
I	2	2	4 (2.6%)
II	37	32	68 (45%)
III	42	35	77 (51%)
IV	1	1	2 (1.4%)
total nodes	82	69	151

**Figure 1:**
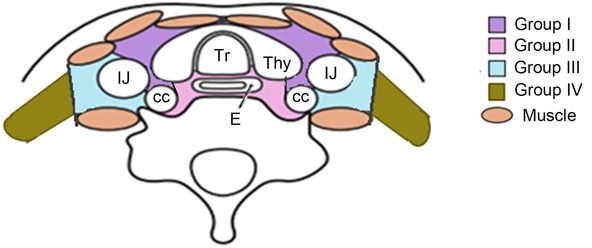
For the lower cervical region is divided into four sub-regions based on the Japan Esophageal Society (JES) and previous literature

**Figure 2:**
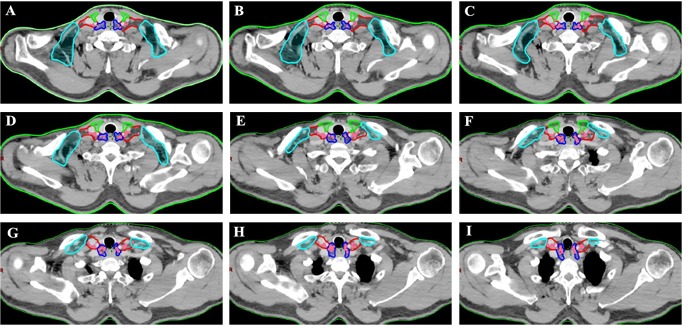
Regions encircled with green line are SubgroupI, those with dark blue line are SubgroupII, those with red line are Subgroup III and those with bright blue line are SubgroupIV Location of lower cervical metastases at presentation. Pink coloration indicates location of nodal disease in patients with lower cervical metastasis.

### Target volume delineation

In our study, the lymph node group with a probability of 10% or more (an empirical cutoff value) of being involved was recommended containing in the CTVn. [12, 14] On the basis of on our findings, 94.4 % (84 of 89 patients) occured the LNM in subgroup II and/or subgroup III region. The anatomic distribution of the 151 LNs indicated that more than 95% of the metastatic LNs was located in the group II and III region. Therefore, the lower cervical group II and III regions had higher rate of LNM, and those subgroup region should receive prophylactic radiation therapy.

**Figure 3:**
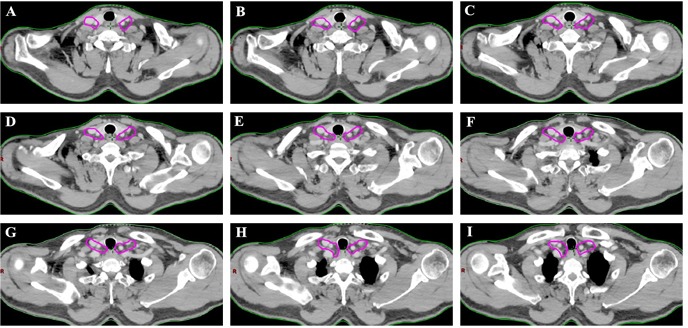
The suggested CTV_n_ for the lower cervical region according to the results The targets should include lower cervical group II and III.

This atlas serves as an available template for target delineation of lower cervical region in the elective treatment of lower cervical nodes in definitive RT/CRT. The suggested CTV_n_ of lower cervical target volumes according to the results are showed in Figure 3.

## DISCUSSION

Precise irradiation of lower cervical lymph node region can minimize treatment toxicity. But the previous studies lack the information of accurate involved LN anatomic distribution. Consequently, it is necessary to find out the risk factors for lower cervical LNM in prophylactic nodal irradiation. It will be helpful for guiding the oncologists in making reasonable decisions on elective nodal irradiation. In addition, we explored the distribution pattern of lower cervical metastatic lymph node, based on image data, and to use these information to propose contours of the lower cervical nodal CTV.

Several studies have report that lymph node metastasis rate in the lower cervical areas was about 17.8%-41.5% in patients with thoracic EC. [15, 16] In the present study, the overall lower cervical LN metastasis incidence was 37.2 % (89 of 239 patients). The metastatic rate of 37.2% in the lower cervical lymph nodes seems to be higher in our study. We think that is mainly because of advanced clinical lesions in most enrolled patients. Moreover, 87% patients in our study were upper and middle thoracic disease, which are high risk factors of lower cervical LNM. In order to determine the suitable indicators for who may benefit from lower cervical region elective irradiation, we analyzed the risk factors for lower cervical LNM. In light of this finding, the tumor location, deeper tumor invasion, involved mediastinal nodal level 1, 2, 4, and 5, and the number of metastatic LN were significant risk factors for LNM in the lower cervical area. The results of multivariate analysis showed that deeper tumor invasion, the mediastinal level 1, 2, and 4 LNM, and the number of LNM were independent risk factors for LN metastasis. Similarly to our result, Tabira and colleagues found that recurrent nerve nodal metastasis and the number of metastatic nodes were associated with cervical lymph node involvement and those factors may be the indicators for the use of three-field dissection in EC. [17] Likely, Li et al. demonstrated that tumor location, depth of tumor invasion, lymphovascular invasion, and paratracheal lymph node metastasis might be helpful in determining the need to perform cervical lymphadenectomy or prophylactic irradiation in individual patient. [18] Taken our study together, these results suggest that for patients with at least one of these factors, prophylactic irradiation of lower cervical areas may confer a benefit.

It was reported that lymph nodes usually locate in the vascular spaces. [19–21] Definition the radiation target volume of lymph node based on vascular rather than bone anatomy could decrease the normal tissue irradiation and minimize treatment related toxicity. In the present research, we gave accurate information for the anatomical spatial distributions and clinical probabilistic incidence of metastatic lymph nodes onto a template CT image according to diagnostic image in EC patients. Based on our analysis, 94.4 % (84 of 89 patients) patients had subgroup II and/or subgroup III region lymph node metastasis, in which 75.3% patients had subgroup III region lymph node metastasis, followed by the subgroup II lymph nodes 69.7% (62 of 89 patients). In contrast, only 5 of 89 patients (5.6 %) with subgroup I and subgroup IV region lymph node metastasis, which suggests that these two subgroups may be spared for irradiation. Besides, total 151 nodes were considered to be clinical metastatic in the lower cervical region in those patients and the atlas showed that the most involved sites of lower cervical LN recurrence were the subgroup II (68 of 151 nodes) and subgroup III (77 of 151 nodes) region, which should be precisely included in the prophylactic volume.

There are limitations to our approach in this study, among them the retrospective nature of the analysis, with which we could not account for all possible biases. Above all, our results in this study regarding the positive lymph nodes are mainly depended on the image rather than the pathological assessments. Therefore, we cannot prove that all of the identified enlarged lymph nodes contain metastatic disease. Furthermore, regarding the imaging method to detect lower cervical lymph node metastasis, literature suggested that CT accuracy for detection of lymph node metastasis is relatively low. [22, 23] Fortunately, some patients included in our study underwent FDG-PET/CT scans,, ultrasound examination and clinical follow-up. In addition, the sample size of our study was not big enough, in all, 151 nodes were considered to be clinical metastatic in the lower cervical region in the 89 patients. But our data showed that the distribution of those involved lymph nodes was highly reproducible, suggesting that it is sufficient to propose the target delineation of lower cervical region.

In conclusion, our findings proposes helpful information for radiation oncologist about the delineation of lower cervical region clinical target volume. Based on the present study, prophylactic irradiating to lower cervical areas is recommended for patients with deeper tumor invasion, the mediastinal level 1, 2, and 4 station LNM and the more number of LNM. The atlas showed that, for the lower cervical area, the subgroup II and III region should be precisely covered in the target volume and the subgroup I and IV may be spared for minimizing the toxicity.

## MATERIALS AND METHODS

### Patient characteristics

Clinical characteristics of esophageal squamous cell carcinoma patients who were initial diagnosis in the Shandong Cancer Hospital and Institute from January 2010 to July 2015 were reviewed. This retrospective review was approved by the institutional review board at the Shandong Cancer Hospital and Institute. All patients had been histologically or cytologically confirmed as ESCC. Esophageal lesions in the reviewed patients were assessed and characterized using examinations that included esophagography, endoscopy, CT imaging, and for some patients, combined positron-emission tomography-CT and endoscopic ultrasonography. Tumor stage and disease grade were classified according to the seven edition of the TNM classification of the American Joint Committee on Cancer (AJCC). The eligible criteria for inclusion in the study were initial diagnosis of ESCC; all patients did not receive anti-tumor treatment; and diagnostic CT scans of the lower cervical region available. Patients were excluded if had received surgery, previous chemotherapy, radiotherapy or have a second primary tumors.

### Diagnostic criteria for lymph node metastasis

Detection of lower cervical lymph node metastasis in our study were included based on the FDG-PET, CT and ultrasound examination of the lower cervical area. All patients were subjected to a contrast-enhanced CT scan of the thorax that extended from the cricoid to the second lumbar vertebra using a maximal slice thickness of 3 mm. All images were reviewed and interpreted by 2 experienced radiological experts. Features supporting a consideration for clinical metastasis included lymph nodes with a short-axis diameter greater than 10 mm; a lymph node with a short axis less than 10 mm combined with hoarseness or vocal cord paralysis; the presence of an infiltrative margin, necrosis, inhomogeneous enhancement; at locations uncommon for reactive nodes; fluorodeoxyglucose-avid (if used). [12, 24, 25]

### The definition of subgroup and node mapping

The mediastinal lymph node nomenclature is based on the new lymph node map published by the IASLC. [26] For the lower cervical region is divided into the left and right sides of the body midline, and each side was further divided into four subgroups based on the Japan Esophageal Society (JES) and Previous literature. [27–30] Subgroup I (No. 100 Superficial lymph nodes of the lower neck ): Cervical pretracheal lymph nodes located in the pretracheal fatty tissue, extending from the hyoid bone superiorly, to the left brachiocephalic vein inferiorly, including the prethyroidal lymph nodes and the prelaryngeal lymph nodes; subgroup II (No. 101 Cervical paraesophageal lymph nodes): Lymph nodes located around the cervical esophagus, including lymph nodes located along the recurrent laryngeal nerve and the cervical paratracheal lymph nodes. The lateral boundary is the medial border of the carotid sheath; subgroup III (No. 104 Lower cervical lymph nodes): Lymph nodes located in the lower cervical fossa, extending from the lower border of the cricoid cartilage superiorly, to the clavicle inferiorly, including the lower internal deep cervical lymph nodes, the medial boundary is the medial border of the carotid sheath; subgroup IV (Posterior cervical lymph nodes level): Lymph nodes extending from lower margin of the cricoid cartilage to the upper border of the manubrium, posterior edge of the sternocleidomastoid. The anatomic diagrammatic sketch of the lower cervical nodes region is shown in Figure 1.

All mapped LN locations were individually reviewed and edited to most precisely represent the corresponding location on the original CT or PET/CT scan by 2 radiologist (Y.L, Y.H) and by 2 radiation oncologists (X.W, J.Y). The nodes were considered to be clinical metastatic in the lower cervical region for all patients were registered by hand drawings from the original CT image onto the template CT image according to methods previously described. [20, 31, 32] The volumetric center of each lymph node was identified and used for mapped on the template images to the best of our ability, and then a volume probability atlas of those nodes was generated. Figure 2 illustrates our LN mapping result for a representative patient. As seen in the atlas, it was displayed on the template image for a visual representation of the density of distribution. Our findings may provide a reference to improve target volume definition in esophageal cancer patients referred for definitive CRT.

### Statistical analysis

All data were analyzed using the SPSS statistical software package (version 20.0, IBM Corp, Armonk, NY, USA). The relationship between other factors and lower cervical lymph node metastasis was examined by univariate and multivariate logistic regression analysis. Factors that were statistically significant in the univariate analysis were entered into multivariate logistic regression analysis. A p value less than 0.05 was considered statistically significant.
